# Flourishing and prosocial behaviors: A multilevel investigation of national corruption level as a moderator

**DOI:** 10.1371/journal.pone.0200062

**Published:** 2018-07-12

**Authors:** Saleh Moradi, Niels Van Quaquebeke, John A. Hunter

**Affiliations:** 1 Department of Psychology, University of Otago, Dunedin, New Zealand; 2 Kühne Logistics University, Hamburg, Germany; University of Warsaw, POLAND

## Abstract

The current psychology literature defines flourishing as leading an authentic life that directs one towards the highest levels of both feeling good and functioning well. Numerous studies show that flourishing relates to a wide array of advantageous personal outcomes. However, the same literature says very little about the social outcomes of flourishing, even though an individual’s pursuit of well-being does not happen in isolation of others. With the present research, we seek to address this void. Specifically, we argue that flourishing, in its psychological conceptualization, does not provide strong moral guidance. As such, flourishing is amoral when it comes to social outcomes such as prosocial behaviors. Drawing on social learning theory, we argue that flourishers’ prosociality is at least somewhat contingent on the moral guidance of their society. To assess this, we tested society’s corruption level as a moderator in the relation between flourishing and prosocial behavior. To that end, we conducted two studies using data from the European Social Survey (ESS), which were collected in 2006 (*N*_*1*_ = 50,504) from 23 countries and in 2012 (*N*_*2*_ = 56,835) from 29 countries. We generally find that corruption at the national level moderates the relation between flourishing and prosocial behaviors (i.e., helping close/distant others, charitable activities). Overall, our study suggests that moral guidance should factor into discussions about flourishing.

## Introduction

Flourishing is commonly understood as the pursuit of an authentic life that directs one towards the highest levels of both hedonic well-being (i.e., feeling good) and eudaimonic well-being (i.e., functioning well) [1,2]. Numerous studies support the idea that a flourishing life relates to a wide array of advantageous outcomes for individuals in terms of physical health, life satisfaction, self-esteem, vitality, academic performance, work productivity, and psychosocial functioning [3–9]. These findings have led to the conclusion that “anything less than flourishing” causes adverse outcomes to the self and society [1]. Indeed, the main argument is that flourishing is not only beneficial to the self, but also contributes to the society’s well-being. As such, flourishing should–at least theoretically–be associated with increased prosocial behavior, i.e., a wide range of voluntary social actions performed to protect or enhance the well-being of others [10,11], including “helping, sharing, donating, co-operating, and volunteering” [12].

Yet, prior research into the relationship between flourishing and prosocial behaviors is restricted in many ways. First, many of the studies that explored the social consequences of flourishing focused mainly on how flourishing affects the welfare of close others [13,14]. However, these findings cannot be easily extended to prosocial behaviors that are exclusively aimed at helping distant others. Indeed, prior research supports that the association between a helper’s well-being and helping behavior is greatly dependent upon the close tie between the helper and the *helpee* [15,16]. Second, the few investigations into the relationship between flourishing and society-improving prosocial behaviors have not always yielded consistent findings. For instance, while Klar and Kasser [17] found a positive association between flourishing and social activation (a specific type of prosocial behavior that aims to improve society through political behavior), Butler and Kern [18], in a more recent study, reported a significant correlation in the reverse direction. Third, in contrast to the findings linking flourishing with higher prosociality, a separate line of research suggests that flourishers may be more engrossed in self-interest convictions than others’ interests [19,20]. The main question is thus: Is flourishing, by itself, enough to predict an individual’s prosocial behavior?

Prosocial behavior, a form of voluntary behavior, is mostly fuelled by a joint function of aptitude, personal motivation, and social norms [21]. While flourishers, endowed with personal resources, seem quite capable of engaging in prosocial behaviors [22,23], no such case can be made regarding their internal motivation to behave prosocially. In fact, on the contrary, previous theoretical discussions suggest that flourishers—propelled to actualize what seems intrinsically desirable to them (e.g., positive emotions, life goals, and personal accomplishments) [24]—are not definitionally motivated to engage in prosocial behavior [25–27]. Lack of a strong internal motivation, then, hint at the critical role of social norms in guiding flourishing individuals willingness to engage in prosocial behavior [21,28,29].

The contradictory findings found in respect of the relationship between flourishing and prosocial behavior thus may be somewhat explained by including the role of social norms [28,29]. Flourishers, much like everyone else in social settings, are subject to what the situational factors expect them to do; these factors, perhaps acting as circumstantial sources of moral guidance, would, then help encourage (or impede) a flourishing individual’s engagement in prosocial behaviors. Specifically, living in an unethical society where selfish practices are perceived to be the business-as-usual discourages individuals to act in prosocial manners (e.g., [30]). Indeed, prior research reveals that corruption at the national level—“a potent and pervasive signal of the ethical tenor of a country” [31]—acts as a decisive negative factor in individuals’ engagement in prosocial behaviors (e.g., [31,32]).

Moreover, building on social learning theory [33], we suggest that the influence of external moral guidance (i.e., what is normatively appropriate) is specifically relevant for flourishing individuals, who are, by definition, not necessarily guided by an internal source of moral guidance. Hence, we argue that the extent to which a society practices and praises moral values affects the relation between flourishing and prosocial behaviors. Particularly, because individuals may incur certain costs for acting against the behavioral expectations of society [34,35], we expect that flourishers would be more likely to abide by social norms in order to preserve or advance personal achievements, and thereby ensure a successful life. More specifically, we assert that the higher a society’s corruption is perceived to be, the less likely a flourishing individual is to engage in prosocial behaviors. Hence, in the present research, we seek to investigate the moderating role of corruption at the national level on the relation between flourishing and prosocial behavior.

Overall, we seek to contribute to the existing literature in two important ways. First, we provide one of the first attempts at turning the research lens away from the flourisher to those who are affected by flourishing (e.g., [13]). In doing so, we contribute to the growing literature on the relationship between flourishing and prosocial behaviors, but with a more nuanced view. Second, by tapping into the emerging discussions over the morality of flourishing [25,27], we maintain that a society’s ethicalness—as a source of situational moral guidance—helps bring out the best (and worst) in flourishing people. As such, we highlight the moderating effect of corruption levels in the relation between flourishing and prosocial behaviors. Ergo, our research sends a key message to those policymakers who, perhaps inspired by the promises of the positive movement [36,37], are considering how to promote personal flourishing as a path toward societal thriving.

### Flourishing

Mostly in the wake of the positive psychology movement [37], scholars’ efforts to define the ultimate level of well-being have mainly pivoted around two prominent understandings—hedonic well-being and eudaimonic well-being. While the former focuses on “feeling good,” the latter emphasizes “functioning well” in various life domains [1,38]. These differences, among others, are usually summarized into a dualism between “feeling good” (i.e., hedonic well-being) and “functioning well” (i.e., eudaimonic well-being) [36]. More recent discussions on the topic [1,39–41] have, however, inspired a general consensus over the fact that the ultimate level of well-being (i.e., flourishing) is *an authentic way of living* that directs toward the highest levels of *both hedonic and eudaimonic well-being* [1,2]. Flourishing thus denotes an active pursuit of positive elements that people pursue for their own sake, rather than a certain psychological end-state [26,42]. Although this general definition has yielded different operationalizations of flourishing, all of them suggest a more or less overlapping set of elements that are conducive to one’s flourishing (for reviews, see [2,43]). These elements include positive emotions, flow, meaning in life, autonomy, achievement, social connectedness, self-acceptance, optimism, and positive relationships [2,5,24,44,45].

Extensive research on well-being, specifically within the positive psychology literature, suggests that flourishers have superior physical health, life satisfaction, self-esteem, vitality, and psychosocial functioning compared to those who pursue either hedonic or eudaimonic well-being [3–9]. Despite these findings, and noting that positive psychologists have always strived to develop positive societies [37], the field has not substantially considered the social outcomes of flourishing: “[P]ositive psychology has demonstrated its usefulness in studying and contributing to individual well-being. The next big challenge for this new field is to help improving [sic] the social and cultural conditions in which people live” [46].

### Flourishing and prosocial behavior: Flourishing is amoral

Ostensibly, as “an optimal range of human functioning” [23], flourishing should foster superiority in social behaviors [44,47]. Indeed, flourishers seem to enjoy many advantages that can drive prosocial behaviors. Flourishers, for instance, experience positive emotions, lead meaningful lives, enjoy positive relationships, and have high self-esteem and competence [2,24]. All of these characteristics are thought to produce abundant personal resources, which further facilitates the *aptitude* necessary for the display of prosocial behavior. Positive emotions, for instance, have been shown to develop one’s cognitive flexibility and breadth; facilitate creative voluntary thoughts and actions; induce outward focus of attention, and build coping skills, resilience, and longevity [22,48]. Likewise, previous research finds that positive emotions drive prosocial attitudes and behaviors such as unselfishness, kindness, relatedness to others, helping, volunteering, donating, and organizational citizenship behavior (see [49] for a meta-review). Based on this literature, and also noting that flourishers have a higher self-esteem and competence in performing their both daily activities and life-long goals, researchers generally assume that flourishers are more capable of engaging in prosocial behaviors than non-flourishers [50].

However, in addition to such aptitude [21], flourishers also need to have a strong *motivation* to engage in prosocial behaviors. Other elements of flourishing may, in part, provide motivation. Having a meaning in life—“belonging to and serving something that you believe is bigger than the self” [24]—may, for instance, help induce self-transcending behaviors that go beyond attending merely to one’s own interest [51]. Indeed, Van Tongeren and colleagues [52] showed that the presence of meaning in life is associated with enhanced self-reported prosocial behavior. In addition, flourishers’ positive relationship with others—“the belief one is cared for, loved, esteemed and valued” [53]—may motivate the expression of reciprocal kindness, warmth, respect and support in relationships between oneself and others [24]. Previous studies indicate that flourishers are more willing to help their close others, which lends support to this argument (e.g., [13,14]).

A closer look at these elements, however, suggests that leading a meaningful life or having positive relationships does not necessarily foster prosocial behaviors, at least in a wider sense. Different examples of prosocial behavior, while having unique characteristics, are all performed with an intention to help or benefit others, i.e., close *and* distant others [10]. As such, prosocial behaviors require a strong attention to others’ interests, and on some occasions, the willingness to prioritize them over and above one’s own personal and in-group interests (e.g., [54]). However, there is nothing inherent to these flourishing elements that would motivate individuals to take interest in the welfare of the broader society. People can develop a life meaning, for example, without specifying what the ‘bigger thing’ can be. It is up to the individual to decide what to gain meaning in life from, whether that be religion, morality, politics, family, nation, or the environment [27]. By the same token, positive relationships with family, friends, compatriots or coreligionists (i.e., in-group members) may not always result in prosocial behaviors toward others’ (i.e., out-group members) well-being [55]. Consequently, it is possible to imagine that individuals could use their strengths to pursue a larger goal that may serve a certain group yet undermine another group’s well-being. Behaviors performed in the name of religious extremism, extreme nationalism, or eco-terrorism, for instance, do not categorize as prosocial behaviors, although they are usually driven by a meaning in life and seek to serve a certain part of society.

Besides, considering that the ultimate goal of flourishing is self-realization [41], flourishers have also been accused of unprincipled self-centeredness, egoism, and narcissism (e.g., [13]). In other words, these conceptual criticisms levy that the striving toward self-actualization is effectively no better than seeking one’s own interest, e.g., egoism (see [56] for a review). These claims have, in fact, received partial support from empirical and conceptual studies that link not only different aspects of well-being (i.e., subjective, psychological, and social), but more particularly, flourishing with narcissistic and self-centered attitudes [19,20].

It follows that flourishing, *per se*, does not seem to innately motivate individuals to engage in prosocial behavior. That is, flourishers can be either prosocial or pro-self. A more comprehensive explanation would include the role of situational factors [21,28,29]. These factors, perhaps acting as circumstantial sources of moral guidance, may encourage or impede a flourisher’s engagement in prosocial behaviors as much as individual differences in moral characteristics [57].

### The moderating effect of corruption as an external source of moral guidance

Researchers argue that social norms, among other situational factors, influence human prosocial actions in powerful ways [58]. Social norms are usually categorized as either descriptive or injunctive [59]. Descriptive norms (i.e., the norms of *is*) simply refer to “what most people do in a given situation”; however, injunctive norms (i.e., the norms of *ought*) denote “behavioral expectations that are backed by (social or material) sanctions” [58]. Prosocial behaviors, being voluntary courses of action, automatically lend themselves better to descriptive norms. As such, descriptive norms are suggested to have an important influence on an individual’s prosociality. Hence, when societies commonly promote the consideration of others’ welfare, their members may be more willing to engage in prosocial behaviors. Conversely, individuals might act more in their own self-interest if they inhabit societies that promote personal gain. Simply put, we are more likely to behave in prosocial ways “if we see others doing so,” and more willing to disregard others’ benefit “if others in [sic] near us are similarly indifferent” [60]. Prior research approves this statement (e.g., [61,62]).

Corruption, then, is usually considered as “a potent and pervasive signal of the ethical tenor of a country” [31]. As such, highly corrupt countries are billboard examples of pro-self, unethical societies with an inordinate prevalence of power being abused for private gain [63], whether in the form of bribing, fraud, theft, or favoritism (nepotism, cronyism, and patronage) [64]. While corruption has been widely studied as a detrimental macroeconomic factor that hinders national economic, political, legal, and societal growth (see [65] for a meta-review), it may also influence individuals’ emotions, attitudes, and choices of action by creating unjust or unfair perceptions [66].

Warren [30], for instance, suggest that corruption erodes people’s confidence that “public decisions are taken for reasons that are publicly available and justifiable,” which further make them “cynical about their own capacities to act on public goods and purposes and [they] will prefer to attend to narrow domains of self-interest they can control.” In that sense, corruption—as an important indicator of egoistic, unethical norms in a society—can also influence individuals’ ethical judgments, attitudes and behaviors [67]. The limited existing research does indeed support that the national level of corruption predicts individuals’ prosocial vs. pro-self orientations and behaviors. Magnus and colleagues [34], for instance, found a positive relationship between higher corruption at the national level and the tolerance of cheating among the students in four countries. Further, data from the World Values Survey, which covers 51 countries, showed that a higher national level of corruption affects individuals’ motivation to participate in the public provisions of goods and services [32]. More recently, in a study spanning 27 nations, Wang and Murnighan [31] noted that corruption at the national level is related to individuals’ increased approval of unethical behaviors and decreased membership in humanitarian and charitable organizations.

Drawing on these findings, we can make the case that corruption, as an indicator of a society’s emphasis on self-interested norms, acts as an important situational factor in deciding individuals’ prosociality. What would suggest its moderating effect on the relation between flourishing and prosociality may, however, be better explained by drawing on social learning theory [33]. According to social learning theory, the acts of observing, learning, conforming with, and imitating what is “normatively appropriate” in a social context is especially relevant for individuals who are not guided by a strong moral compass (see also [57]). These people tend to follow a ‘social proof’ strategy—an assumption that others’ actions in a given situation reflect the normatively right behavior—rather than ‘think through’ the ethical course of action by following their personal convictions [60]. Similarly, we argue that flourishing individuals, who are mainly driven by a moral-free form of self-realization, would find less reason to behave prosocially while living in a corrupt society where considering others’ well-being is not a social norm—especially since prosocial behavior would be nonconformist in such societies and could thus create certain costs for the individual [34,35].

In short, we argue that flourishers’ prosociality is somewhat contingent on the ethical norms of the society they live in. Hence, we propose a model in which the national level of corruption moderates the relationship between flourishing and prosocial behavior. Particularly, we argue that flourishers would be less willing to consider others’ well-being when living in a country with a higher level of corruption. More specifically, we argue that high corruption deters flourishing individuals from engaging in prosocial behaviors. Thus, we advance the following hypothesis:

*Hypothesis 1*: Corruption at the national level moderates the association between flourishing and prosocial behavior. Specifically, the effect of flourishing on prosocial behavior will be more pronounced when national level of corruption is low versus high.

### Present studies

We tested our hypothesis across two large, cross-national samples obtained from the European Social Survey (ESS) dataset. ESS originally emerged as a means of providing rigorous comparative analysis across European countries and over time [68]. The ESS data is gathered via individual face-to-face interviews, and its target population covers individuals above 15 years of age who are residents within private households, regardless of nationality or citizenship, language or legal status. The ESS is generally considered to be a prominent source of cross-national data with strong validity and reliability, with a mean response rate of over 60% [69]. It should be noted that ESS, while not offering longitudinal data, provides information about trends over time in people’s underlying status, values, opinions, attitudes, and behaviors in various personal, social and political domains. ESS has been run biennially since 2002 and has produced seven rounds of data since then. However, the ‘Personal and Social Well-being’ module that includes the ‘flourishing’ items have only appeared twice: in ESS 3, collected in 2006/2007, and ESS 6, collected in 2012/2013. Consequently, we were confined to using the data collected in these two rounds to test our model.

Furthermore, we followed current recommendations in the field with regard to including control variables (e.g., [70,71]). Previous studies indicate a theoretical linkage and significant empirical association between age, gender, and also national levels of GDP per capita, income inequality, and individualism with both the predictor (i.e., flourishing) and the outcome variable (i.e., prosocial behaviors) [31,47,66,72–75]. Thus, we controlled for their main effects throughout both Studies 1 and 2. We obtained the information on GDP per capita (i.e., GDP per capita based on purchasing power parity) and income inequality (i.e., GINI Index) from the World Bank. National levels of individualism were captured using the Individualism Index [76]. However, a preliminary analysis showed no main effect for gender and the Individualism Index: Regardless of their presence or absence, we found identical results. Thus, to maximize statistical power and offer the most interpretable results, we report the findings without controlling for gender and the Individualism Index.

## Study 1

### Method

#### Participants and procedure

For this study, we used the 2006 European Social Survey (ESS 3), which offered a dataset featuring 50,504 participants from 23 countries. Prior to the analysis, we weighted the data using standard ESS recommended techniques to ensure that: (a) the sample in each country was representative of its population; and (b) each country was represented in proportion to its population size. The data, alongside the extensive documentation about the weighting process, are freely available from the ESS website using the search phrases: “Data and Documentation by Year”, “Round 3 (2006)” and “More files and documents” (http://www.europeansocialsurvey.org/). A more complete description of the study sample’s characteristics is presented in Table 1.

**Table 1:** Descriptive statistics and correlations in Study 1.

	*N*	*Female %*	Age	CPI^1^ 2006	GDP per capita 2006 (in USD)	GINI Index 2005–2007	Individualism Index	Flourishing	Charitable activities	Helping distant others	*r* (flourishing and charitable activities)	*r* (flourishing and helping distant others)	*r* (charitable activities and helping distant others)
Austria	695	51.8	46.2	8.6	37455.51	29.59	55	0.207	2.608	3.705	.205***	.245***	.466***
Belgium	871	51.5	46.3	7.3	35406.63	28.26	75	0.107	1.984	3.334	.117**	.099**	.309***
Bulgaria	667	52.3	46.6	4.0	11377.81	35.73	30	-0.265	1.114	2.137	.098*	.189***	.232***
Switzerland	627	51.4	46.6	9.1	44951.63	34.50	68	0.335	2.833	3.776	.119**	.146***	.359***
Cyprus	63	50.8	43.9	5.6	30496.04	31.13	-	0.220	1.758	2.814	.114	.295*	.363**
Germany	7079	51.7	47.7	8.0	34261.47	32.78	67	0.071	2.507	3.899	.218***	.221***	.372***
Denmark	441	50.8	47.3	9.5	37327.17	27.08	74	0.415	2.195	4.078	.148**	.163**	.254***
Estonia	114	55.3	45.4	6.7	19269.08	33.75	60	-0.030	1.384	2.345	.133	.077	.374***
Spain	3744	50.8	46.1	6.8	30832.97	32.67	51	0.119	1.918	2.627	.122***	.120***	.389***
Finland	435	51.7	47.1	9.6	34382.80	28.02	63	0.244	2.160	3.487	.110*	.140**	.205***
France	4982	52.3	46.3	7.4	32543.36	29.92	71	0.036	2.108	3.284	.097***	.072***	.351***
United Kingdom	6173	51.5	46.2	8.6	34332.30	34.84	89	0.099	2.169	3.105	.137***	.135***	.389***
Hungary	900	53.5	46.3	5.2	18230.04	30.00	80	-0.119	1.385	2.353	.084*	.129***	.390***
Ireland	337	50.5	42.6	7.4	44246.45	32.73	70	0.231	2.301	3.220	.157**	.136*	.410***
Netherlands	1335	50.9	45.9	8.7	40620.76	30.76	80	0.152	2.662	3.269	.132***	.141***	.459***
Norway	373	50.9	46.1	8.8	54110.88	27.29	69	0.246	2.675	3.623	.070	.146**	.281***
Poland	3197	52.4	43.8	3.7	15150.90	34.71	60	-0.081	1.272	2.458	.105***	.154***	.283***
Portugal	893	52.1	46.4	6.6	24669.57	38.06	27	-0.034	1.655	1.880	.076*	.058^†^	.577***
Russia	12075	54.6	44.3	2.5	14916.19	41.54	39	-0.172	1.377	2.307	.088***	.107***	.384***
Sweden	837	50.8	46.8	9.2	37439.84	26.47	71	0.227	1.844	3.906	.080*	.129***	.169***
Slovenia	172	51.2	45.7	6.4	25777.99	24.48	27	0.045	1.959	4.058	.130^†^	.216**	.315***
Slovakia	449	51.4	43.2	4.7	18875.53	27.71	52	-0.140	1.445	2.872	.124**	.173***	.325***
Ukraine	4046	55.4	45.4	2.8	7184.20	29.79	25	-0.133	1.536	2.407	.159***	.139***	.495***
Total	50504	52.7	45.8	5.830	29733.004	31.383	59.227	-0.004	1.879	2.932	.177***	.184***	.418***

Notes. ^1^Corruption Perception Index (higher values = lower corruption).

^†^*p* < .10.

*p < .05.

**p < .01.

***p < .001.

#### Measures

**Flourishing.** To measure flourishing, we followed Huppert and So’s [2] instructions to utilize 10 items from the ESS 3. Their operationalization of flourishing combined three main factors: positive characteristics (i.e., emotional stability, optimism, resilience, self-esteem, and vitality), positive functioning (i.e., competence, engagement, meaning, and positive relationships) and positive emotion. Example items include: “I am always optimistic about my future” (optimism); “(In the past week) I had a lot of energy” (vitality); “Most days I feel a sense of accomplishment from what I do” (competence); “There are people in my life who really care about me” (positive relationships), and “Taking all things together, how happy would you say you are?” (positive emotions). Considering the inconsistency of Likert scales used for different items (1 = strongly disagree, 5 = strongly agree, for seven items; 1 = none or almost none of the time, 4 = all or almost all of the time, for two items; and 0 = extremely unhappy, 10 = extremely happy, for one item), we derived an overall flourishing score by calculating an average score among the standardized items for each subscale, and then aggregating those scores (also see [77]). Cronbach’s alpha was .74.

**Prosocial behavior.** Two questions in the ESS 3 concern prosocial behaviors: The first question asks about respondents’ involvement in charitable activities (i.e., "In the past twelve months, how often did you get involved in work for voluntary and charitable organizations?"). The second question measures respondents’ engagement in helping distant others (i.e., "Not counting anything you do for your family, in your work, or within voluntary organizations, how often, in the past twelve months, did you actively provide help for other people?"). In order to be consistent with the definition of prosocial behavior (e.g., [10]), we labeled these questions as representative of *charitable activities* and *helping distant others* (cf., [78,79]). In the present study, we reverse-coded both questions, which were originally rated on a 6-point scale ranging from 1 (at least once a week) to 6 (never).

**Corruption.** To assess the extent of corruption within a country, we used the Corruption Perception Index (CPI), which has been published yearly since 1995 by Transparency International. Prior research has used the CPI in intercultural studies of corruption [66,80] and found it to be a valid and reliable measure of corruption [63,81]. The CPI draws on multiple sources provided by independent expert and business institutions, who complete several surveys/polls to provide a global index of the corruption in each country. Based on these sources, the CPI for each country is then calculated as an arithmetic mean of the level of corruption. Experts and institutions are asked to assess, for instance, “to what extent are public officeholders who abuse their positions prosecuted or penalized?”, “do whistleblowers, anti-corruption activists, investigators, and journalists enjoy legal protections that make them feel secure about reporting cases of bribery and corruption?”, and “in your country, how common is diversion of public funds to companies, individuals or groups due to corruption?”. See the Transparency International website for a complete listing of the surveys used, the characteristics of the sample/survey, the administrating organizations, and extensive documentation (http://www.transparency.org/research/cpi/).

The range of the CPI reported by Transparency International in 2006 (i.e., the same year of the ESS 3 data collection) was 1 to 10, with higher scores indicating lower corruption. The CPI for each country included in ESS 3 is presented in Table 1.

### Results and discussion

Table 1 presents the descriptive statistics, sample sizes, national means, and correlations among individual-level variables for each country. In general, flourishing was positively correlated with both charitable activities and helping behavior. However, our analyses affirmed only a weak correlation between flourishing and prosocial behaviors across the samples from different countries, ranging between *r* = .070 to .218 for charitable activities, and *r* = .058 to .295 for helping distant others (see Table 1).

Multi-level modeling (MLM) was used to test the interaction effect of flourishing (i.e., individual-level variable) and corruption (i.e., national-level variable) on prosocial behavior. For each outcome variable (i.e., charitable activities and helping behavior), we tested a progression of models using maximum likelihood estimation; model fit was evaluated using the likelihood ratio test, according to Hayes’ [82] recommendations. Moreover, following Enders and Tofighi’s [83] guidelines, we group-mean centered individual-level variables (i.e., flourishing and participants’ age) and grand-mean centered national-level variables (i.e., CPI, GDP per capita, and GINI Index). The progression of models tested was as follows: Model 1 (i.e., random intercept-only model) assessed whether countries differ from each other, on average, on the outcome variable. It also estimated the degree of nonindependence in the outcome variable across individuals—interclass correlation (ICC). Model 2 assessed the relation between flourishing and the outcome variable. Model 3 added the effect of participants’ age, as an individual-level control variable, to the previous model. Model 4 assessed between-country differences in the relation between flourishing and the outcome variable, while controlling for participants’ age. Model 5 added the main effect of CPI, GDP per capita, and the GINI Index, as national-level variables, to the previous model. Finally, Model 6 added the cross-level interaction effect of flourishing and CPI to the previous model.

To estimate the effect sizes of our MLM, we followed other scholars’ suggestions in reporting both Pseudo *R*^*2*^(S&B) and the cross-level interaction’s explanatory power—the slope variance explained by corruption at the national level (see [84,85] for equations and detailed discussions about effect size estimation in MLM).

#### Flourishing, CPI and charitable behaviors

Table 2 presents the MLM results regarding the relation between flourishing, CPI and charitable behaviors in Study 1. The likelihood ratio test was used to compare the deviances (i.e., − 2LL) between Model 1 and a model without the random component of the intercept [82]. In Model 1, − 2LL = 164,999.986. Without the random component of the intercept, − 2LL = 169,259.789, with a difference of 169,259.789–164,999.986 = 4,259.803. These two models differed by a single degree of freedom, for *χ*^2^(1) = 4,259.803, *p* < .001, which suggests that participants from different countries vary significantly on their average participation in charitable activities. Furthermore, with an ICC of 0.116, it can be said that 11.6% of the total variance of charitable activities was accounted for by country. Models 2–3 consistently indicated the positive association between flourishing and charitable activities, even when controlling for the main effect of participants’ age.

**Table 2:** Parameter estimates for the six models examining the relation of charitable activities on flourishing and corruption in Study 1.

		Model 1	Model 2	Model 3	Model 4	Model 5	Model 6
Fixed components							
Intercept	*γ*_00_	1.937***	1.932***	1.935***	1.939***	1.971***	1.894***
Flourishing (F)	*γ*_10_		.321***	.333***	.374***	.351***	.305***
Age	*γ*_20_			.002***	.001***	.002***	.002***
CPI	*γ*_01_					-.012	.069***
GDP per capita	*γ*_02_					.000*	.000^†^
GINI Index	*γ*_03_					.005	.008
F × CPI	*γ*_11_						.061*
Variance of random components							
	*τ*_00_	.288**	.289**	.290**	.286**	.120*	.086**
	*τ*_11_				.114**	.078*	.050*
	*τ*_01_				.054*	.056*	.034*
	*σ*^2^	2.191	2.156	2.153	2.138	2.138	2.138
Model fit							
Model deviance (–2LL)		164999.986	164258.663	163353.464	163049.570	163034.261	163027.964
Model Δ*χ*^2^			741.323***	905.199***	303.894***	15.309**	6.297*
*Δdf*			1	1	2	3	1
Pseudo *R*^*2*^		0	.014	.015	.022	.089	.103
Explanatory Power							.358

Notes. CPI = Corruption Perception Index (higher values = lower corruption).

^†^*p* < .10.

*p < .05.

**p < .01.

***p < .001.

Model 4 added a random component on the relation between flourishing and charitable activities, such that the slopes predicting charitable activities from flourishing were allowed to vary freely between countries. The model showed a significant improvement of fit to the data, which indicates that the relation between flourishing and charitable activities differs between countries. The significance of Model 4 further justifies the investigation of a cross-level moderation effect. Model 5 indicated a non-significant influence of national-level CPI on charitable activities after controlling for individual-level differences on flourishing and age, and national-level differences on GDP per capita and GINI Index. Model 6, however, supported that national-level CPI moderates the relation between flourishing and charitable activities. The significance of the positive interaction term means that (a) the effect of flourishing on charitable activities depends on CPI and (b) the coefficient for flourishing is larger in countries with higher CPI (i.e., less corruption). Particularly, the calculated explanatory power revealed that the moderating effect of national-level CPI accounted for 35.8% of the slope variance in the relation between flourishing and charitable activities.

**Fig 1:**
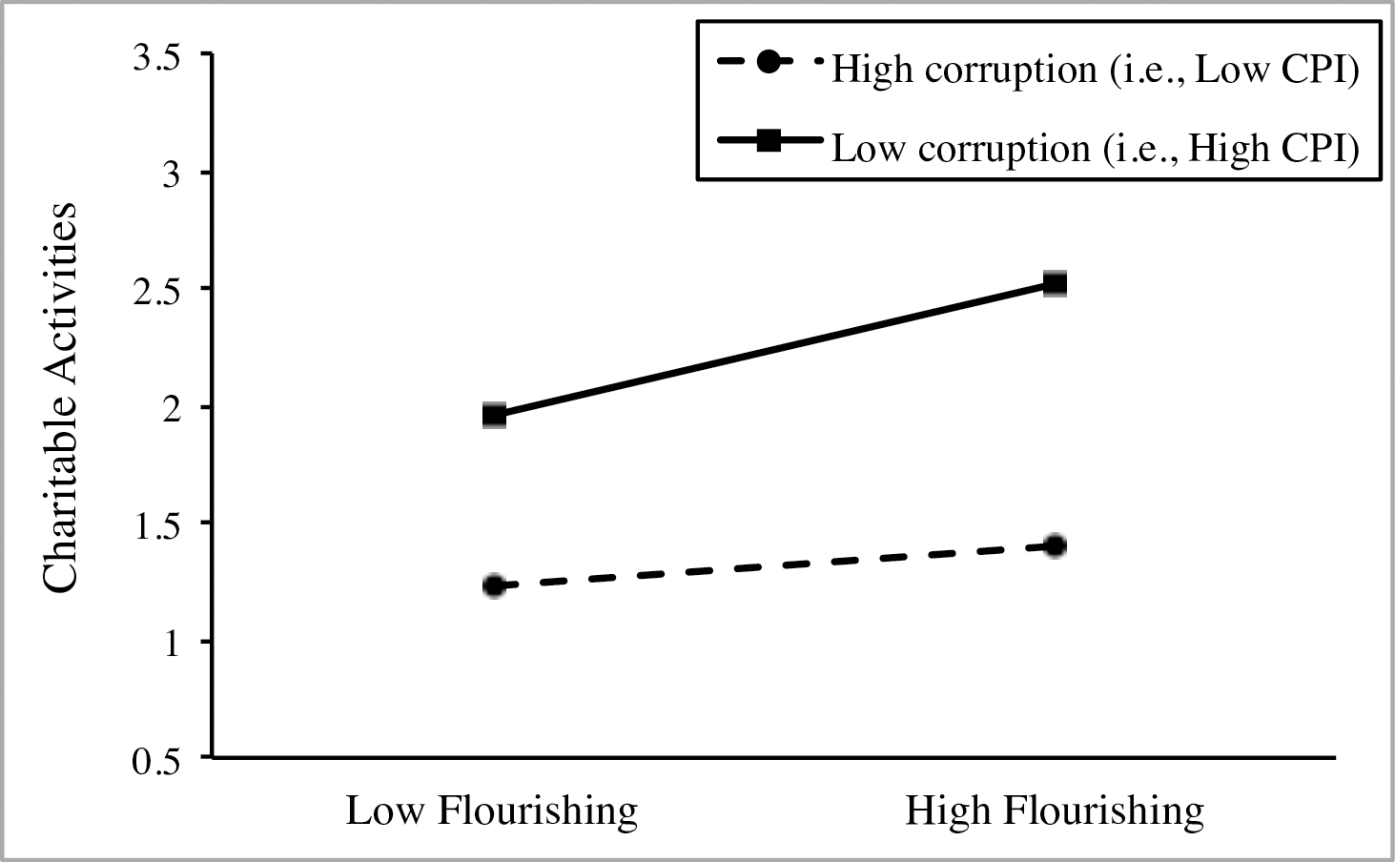
Cross-level interaction between flourishing and CPI on charitable activities in Study 1.

Fig 1 depicts the moderation, which was produced using the tools provided by Preacher, Curran, and Bauer [86]. An additional simple slope analysis showed a stronger positive association between flourishing and charitable activities in countries high in CPI (i.e., less corruption; simple slope *B* = .470, *p* < .001) in comparison to countries low in CPI (i.e., high corruption; simple slope *B* = .144, *p* = .160, not significant).

#### Flourishing, CPI and helping distant others

Following similar steps as above, we utilized MLM to examine the relation between flourishing, CPI and helping distant others (see Table 3). The likelihood ratio test indicated a − 2LL difference of 184,497.068–179,958.799 = 4,538.269 between Model 1 and a model without the random component of the intercept. There was a single degree of freedom difference between the two models, for *χ*^2^(1) = 4,538.269, *p* < .001, which suggests that participants from different countries vary significantly in terms of how much they help distant others, on average. The ICC was 0.123, indicating that 12.3% of the total variance of helping distant others was due to between-country variation. Model 2 showed that flourishing is positively associated with helping distant others, and Model 3 indicated that this association remains significant after controlling for participants’ age.

**Table 3:** Parameter estimates for the six models examining the relation of helping distant others on flourishing and CPI in Study 1.

		Model 1	Model 2	Model 3	Model 4	Model 5	Model 6
Fixed components							
Intercept	*γ*_00_	3.024***	3.018***	3.009***	3.009***	3.092***	3.018***
Flourishing (F)	*γ*_10_		.413***	.382***	.409***	.378***	.352***
Age	*γ*_20_			-.005***	-.005***	-.005***	-.005***
CPI	*γ*_01_					-.023	.055
GDP per capita	*γ*_02_					.000^†^	.000^†^
GINI Index	*γ*_03_					-.046*	-.044*
F × CPI	*γ*_11_						.042*
Variance of random components							
	*τ*_00_	.458**	.464**	.466**	.460**	.152*	.127**
	*τ*_11_				.097*	.060*	.045*
	*τ*_01_				.029*	.031*	.021*
	*σ*^2^	3.269	3.212	3.208	3.199	3.199	3.199
Model fit							
Model deviance (–2LL)		179958.799	179154.935	178146.705	178037.261	178017.809	178013.177
Model Δ*χ*^2^			803.864***	1008.230***	109.444***	19.452***	4.632*
*Δdf*			1	1`	2	3	1
Pseudo *R*^*2*^		0	.014	.015	.018	.101	.108
Explanatory Power							.250

Notes. CPI = Corruption Perception Index (higher values = lower corruption).

^†^ *p* < .10.

* *p* < .05.

** *p* < .01.

*** *p* < .001.

**Fig 2:**
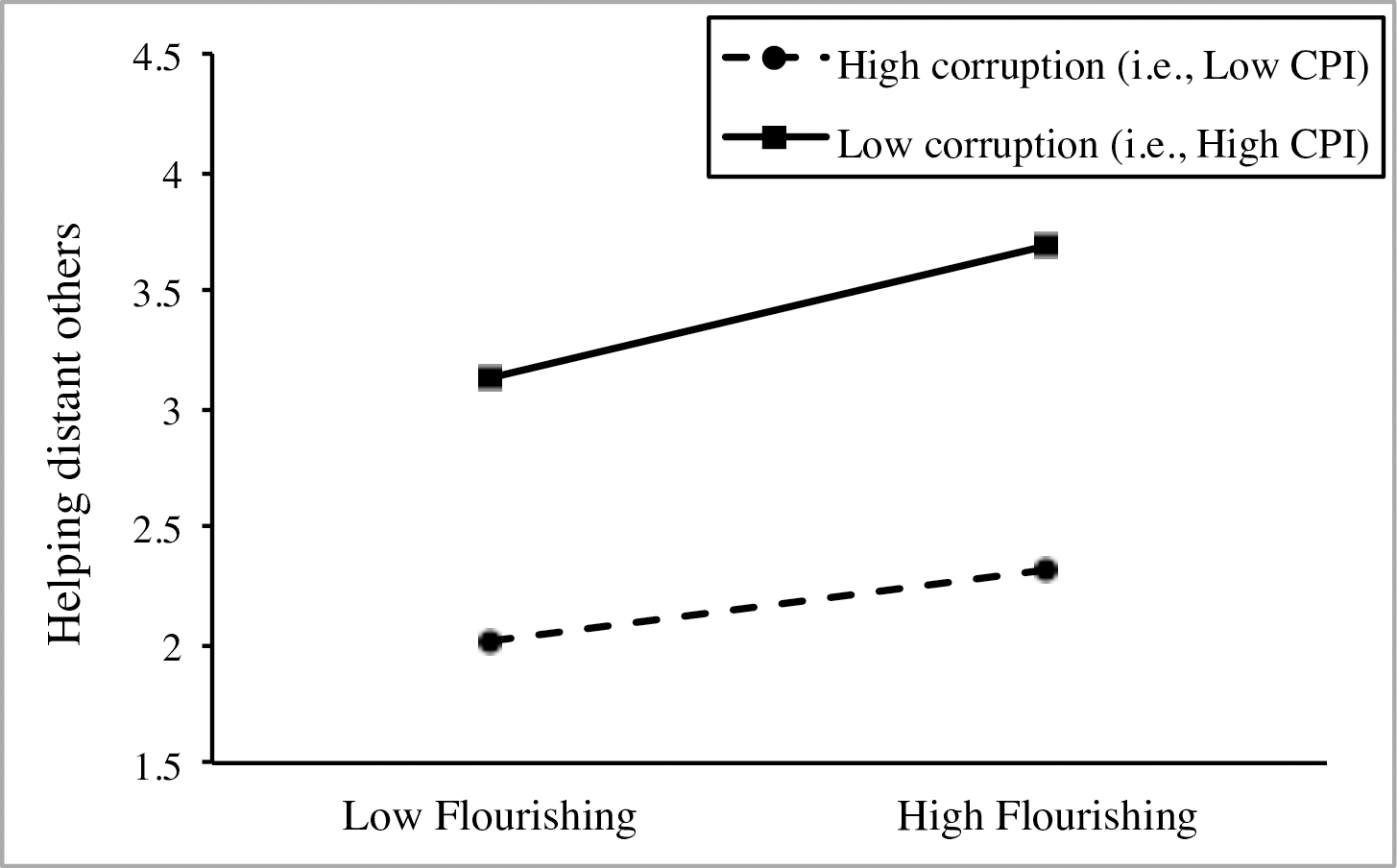
Cross-level interaction between flourishing and CPI on helping distant others in Study 1.

Model 4 indicated that the relation between flourishing and helping distant others differs between countries, justifying the investigation of a cross-level moderation effect. Model 5 showed that the main effect of national-level CPI on helping distant others was not significant while controlling for individual-level differences on flourishing and age, and national-level differences on GDP per capita and GINI Index. Finally, Model 6 supported the moderating effect of national-level CPI on the relation between flourishing and helping distant others. The significance of the positive interaction term suggests that (a) the effect of flourishing on helping distant others depends on CPI and (b) the coefficient for flourishing is larger in countries with higher CPI (i.e., less corruption), as depicted in Fig 2. In particular, the calculated explanatory power indicated that the moderating effect of national-level CPI accounted for 25.0% of the slope variance in the relation between flourishing and helping distant others. Further simple slope analysis showed a stronger positive association between flourishing and helping distant others in countries high in CPI (i.e., low corruption, simple slope *B* = .473, *p* < .001) in comparison to countries low in CPI (i.e., high corruption, simple slope *B* = .245, *p* = .006).

Collectively, these findings support our Hypothesis 1: The cross-level interaction effect of flourishing and corruption was significantly associated with prosocial behaviors (i.e., charitable activities and helping distant others). Particularly, flourishing showed a stronger positive effect on engagement in charitable activities and helping distant others for individuals who were living in less corrupt countries, as we predicted.

## Study 2

The results of Study 1, using the ESS 3 data, generally show that corruption at the national level affects the relation between flourishing and engagement in prosocial behaviors. In particular, it seems that flourishing individuals living in more corrupt countries are less likely to engage in prosocial behaviors, i.e., charitable activities and helping distant others, as predicted in Hypothesis 1. With Study 2, we aimed to reassess Hypothesis 1 using the ESS 6 data. While the item measuring ‘charitable activities’ was the same in this wave of ESS, the item measuring ‘helping distant others’ in ESS 3 was replaced with an item asking about participants’ ‘helping close others’ in ESS 6. This provided us a unique opportunity to test our moderation model with an alternative example of prosocial behavior that specifically targets close others. Thus, we sought to elaborate whether corruption, as a contextual source of moral guidance, moderates the relation between flourishing and helping close others—and in what direction, if so.

Pursuing positive relationships with close others is usually considered a definitional element of flourishing (see above): Both theoretical and empirical accounts note that flourishers are internally motivated to help and support their close others [13,24]. Moreover, while we acknowledge that corruption normalizes social practices that are directed toward “private gain”, even individuals low in moral characteristics tend to consider their close others in their circle of moral regard [55,87]. Hence, helping close others, unlike the other types of prosocial behavior that target distant others, is rarely considered to be an example of other-regard and thus may be less directed by moral guidance. If so, is it possible to say that corruption (e.g., a source of moral guidance) does not influence the relation between flourishing and helping close others?

The answer to this question requires a closer look at the definition of corruption. Corruption, by definition, includes a favoritism (nepotism, cronyism, and patronage) dimension that, in turn, entails individual behavior “which deviates from the formal duties of a public role because of private-regarding (personal, close family, private clique) pecuniary or status-gain” [88]. Higher corruption levels thus somewhat ‘regularize’ or ‘justify’ offers of egoistically motivated help to in-group members (i.e., close others) [64]. Moreover, corruption creates perceptions of injustice, grievance, anger and retaliation [31], which may further motivate individuals to guard their in-group members’ benefits. As such, we argue that flourishing individuals living in a highly corrupt country not only find helping close others to be congruent with (rather than against) the social norms, but may even see such actions as crucial for protecting their close others’ benefits. Accordingly, we hypothesize:

*Hypothesis 2*: Corruption at the national level moderates the association between flourishing and helping close others. Specifically, the effect of flourishing on helping close others will be more pronounced when national level of corruption is high versus low.

### Method

#### Participants and procedure

We used the 2012 European Social Survey (ESS 6), which offered a dataset of 56,835 participants from 29 countries. Preceding the analysis, we weighted the data using standard ESS recommended techniques to ensure that: (a) the sample in each country was representative of its population; and (b) each country was represented in proportion to its population size. A more complete description of the study sample characteristics is presented in Table 4. The data and extensive documentation are freely available from the ESS website using the search phrases: “Data and Documentation by Year”, “Round 6 (2012)” and “More files and documents” (http://www.europeansocialsurvey.org/).

**Table 4:** Descriptive statistics and correlations in Study 2.

	*N*	*Female %*	Age	CPI^1^ 2012	GDP per capita 2012 (in USD)	GINI Index 2012	Individualism Index	Flourishing	Charitable activities	Helping close others	*r* (flourishing and charitable activities)	*r* (flourishing and helping close others)	*r* (charitable activities and helping close others)
Albania	223	50.9	42.8	34	10526.87	28.96	20	-0.031	1.656	5.091	.195**	.373***	.114^†^
Belgium	921	51.6	48.0	75	42354.63	27.59	75	0.006	2.008	5.073	.141***	.343***	.075*
Bulgaria	635	51.8	48.2	36	16208.32	36.01	30	-0.150	1.215	5.194	.154***	.459***	.059
Switzerland	676	52.5	46.6	86	57590.70	31.64	68	0.263	2.808	5.304	.041	.376***	.007
Cyprus	72	52.8	44.1	66	31877.64	34.31	-	0.125	1.878	5.391	.201^†^	.329**	.097
Czech Republic	896	51.0	46.2	49	29047.25	26.13	58	-0.112	1.506	4.754	.125***	.444***	.168***
Germany	7101	51.4	48.7	79	43564.15	30.01	67	0.206	2.780	5.403	.148***	.347***	.033**
Denmark	459	50.8	47.4	90	44511.33	29.08	74	0.320	2.360	5.289	.162***	.340***	.042
Estonia	113	54.4	47.0	64	26022.47	33.15	60	-0.004	1.583	4.995	.156	.457***	.072
Spain	3917	51.1	46.9	65	32082.30	35.89	51	0.003	2.376	5.341	.113***	.327***	.053**
Finland	451	51.4	48.1	90	40620.18	27.12	63	0.140	2.067	4.985	.091^†^	.369***	.056
France	5319	52.2	47.6	71	37645.31	33.10	71	-0.001	2.063	5.267	.082***	.297***	.043**
United Kingdom	5217	51.8	46.5	74	37477.80	32.57	89	0.004	2.338	5.261	.133***	.322***	.029*
Hungary	852	53.2	46.7	55	22997.75	30.55	80	-0.170	1.420	5.072	.214***	.495***	.124***
Ireland	359	46.5	44.5	69	46552.98	32.52	70	0.098	2.330	5.168	.160**	.440***	.121*
Israel	563	50.2	41.5	60	31750.63	42.78	54	0.135	2.103	5.207	.116**	.356***	.077^†^
Iceland	25	50.0	43.8	82	40418.44	26.94	60	0.194	2.438	5.365	.133	.311	.094
Italy	5229	52.1	48.8	42	36237.11	35.16	76	-0.025	2.121	5.192	.057***	.346***	.084***
Lithuania	256	54.7	46.0	54	24647.99	35.15	60	-0.147	1.424	4.975	.200**	.494***	.143*
Netherlands	1383	50.7	46.7	84	46707.27	27.99	80	0.149	2.898	5.188	.128***	.299***	.052^†^
Norway	406	50.2	45.5	85	65380.25	25.90	69	0.161	2.709	5.202	.135**	.342***	.018
Poland	3272	50.6	46.1	58	23832.73	32.39	60	0.076	1.469	5.170	.135***	.426***	.093***
Portugal	898	53.2	48.2	63	26454.10	36.04	27	-0.089	1.594	4.890	.124***	.399***	.056^†^
Russia	12139	54.7	43.9	27	25316.64	41.59	39	-0.154	1.608	4.658	.095***	.472***	-.006
Sweden	790	50.5	46.9	88	44724.97	27.32	71	0.150	1.954	5.350	.130***	.379***	.050
Slovenia	176	50.6	47.0	61	28841.92	25.59	27	0.159	1.962	5.429	.173*	.467***	.073
Slovakia	457	52.2	44.5	46	26647.42	26.12	52	-0.082	1.755	5.145	.172***	.425***	.117*
Ukraine	3892	54.8	44.6	33	8475.47	24.74	25	-0.101	1.453	5.023	.088***	.395***	.040*
Kosovo	135	48.9	37.5	37	8541.31	29.40	-	0.133	1.660	5.278	.165^†^	.412***	.121
Total	56835	52.4	46.4	55.869	33001.928	31.232	58.370	-0.004	2.029	5.101	.149***	.410***	.086***

Notes. ^1^Corruption Perception Index (higher values = lower corruption).

^†^ *p* < .10.

* *p* < .05.

** *p* < .01.

*** *p* < .001.

#### Measures

**Flourishing.** We used the same procedure as in Study 1 to measure flourishing. The only differences between the two measures referred to the items used to assess positive relationships and engagement. While Study 1 (i.e., ESS 3) measured positive relationships and engagement with the items “There are people in my life who really care about me” and “I love learning new things”, these same items did not exist in ESS 6. Hence, following Ruggeri and colleagues [77], these items were accordingly replaced by “To what extent do you receive help and support from people you are close to when you need it?” and “How much of the time are you absorbed in what you are doing?” from ESS 6. Cronbach’s alpha was .73.

**Prosocial behavior.** The ESS6 uses the same question as the ESS 3 (Study 1) for measuring *charitable activities* as a form of prosocial behaviors. The question reads: "In the past twelve months, how often did you get involved in work for voluntary and charitable organizations?" Again, we reverse-coded the questions that were originally rated on a 6-point scale, ranging from 1 (at least once a week) to 6 (never). The question measuring ‘helping distant others’ in ESS 3 was, however, replaced in ESS6 with an item asking about participants’ engagement in helping close others, i.e., "To what extent do you provide help and support to people you are close to when they need it?" The question was rated on a 7-point scale ranging from 0 (not at all) to 6 (completely).

**Corruption.** For corruption, we used Transparency International’s 2012 CPI (i.e., the same year of ESS 6 data collection). While the reported CPI in 2006, used in Study 1, scored on a 0–10 scale, the CPI 2012 rated each country’s corruption level on a scale of 0 to 100. Again, higher scores indicate a lower national corruption level. The CPI for each country included in ESS 6 is presented in Table 4.

### Results and discussion

Table 4 presents the descriptive statistics, sample sizes, national means, and correlations among individual-level variables for each country. In general, within countries, flourishing was positively correlated with both charitable activities and helping close others. However, as somewhat expected, the correlation between flourishing and helping close others (*r* = .297 to .495) was much stronger than the correlation between flourishing and charitable activities (*r* = .041 to .214) across the samples from different countries. Moreover, the analyses indicated a very weak positive correlation between helping close others and charitable activities (*r* = *-*.006 to .168).

Following the same progression of models as in Study 1, we used multi-level modeling to test the interaction effect of flourishing (i.e., individual-level variable) and CPI (i.e., national-level variable) on each outcome variable (i.e., charitable activities and helping close others).

#### Flourishing, CPI and charitable activities

The results of the MLM examining the relation between flourishing, CPI and charitable activities in Study 2 are presented in Table 5. The likelihood ratio test indicated a − 2LL difference of 192,627.046–188,314.430 = 4,312.616 between Model 1 and a model without the random component of the intercept. There was a single degree of freedom difference between the two models, for *χ*^2^ (1) = 4,312.616, *p* < .001, which suggests that participants from different countries vary significantly on their average participation in charitable activities. The ICC was .08, indicating that 8.0% of the total variance of charitable activities was due to between-country variation. Model 2 provided that flourishing is positively associated with charitable activities, and Model 3 indicated that this association remains significant after controlling for participants’ age.

**Table 5:** Parameter estimates for the six models examining the relation of charitable activities on flourishing and CPI in Study 2.

		Model 1	Model 2	Model 3	Model 4	Model 5	Model 6
Fixed components							
Intercept	*γ*_00_	1.933***	1.933***	1.934***	1.943***	2.056***	2.024***
Flourishing (F)	*γ*_10_		.289***	.285***	.301***	.296***	.282***
Age	*γ*_20_			-.000	-.001	-.001	-.001^†^
CPI	*γ*_01_					-.003	.006
GDP per capita	*γ*_02_					.000**	.000*
GINI Index	*γ*_03_					.010	.011
F × CPI	*γ*_11_						.006***
Variance of random components							
	*τ*_00_	.213**	.217**	.218**	.216**	.104**	.085**
	*τ*_11_				.048*	.031*	.013
	*τ*_01_				.018*	.018*	.006^†^
	*σ*^2^	2.437	2.411	2.410	2.404	2.404	2.404
Model fit							
Model deviance (2LL)		188314.430	187746.097	187071.506	186951.011	186929.715	186918.248
Model Δ*χ*^2^			568.333***	674.591***	120.495***	21.296***	11.467***
*Δdf*			1	1	2	3	1
Pseudo *R*^*2*^		0	.008	.008	.011	.054	.061
Explanatory Power							.581

Notes. CPI = Corruption Perception Index (higher values = lower corruption).

^†^ *p* < .10.

* *p* < .05.

** *p* < .01.

*** *p* < .001.

**Fig 3:**
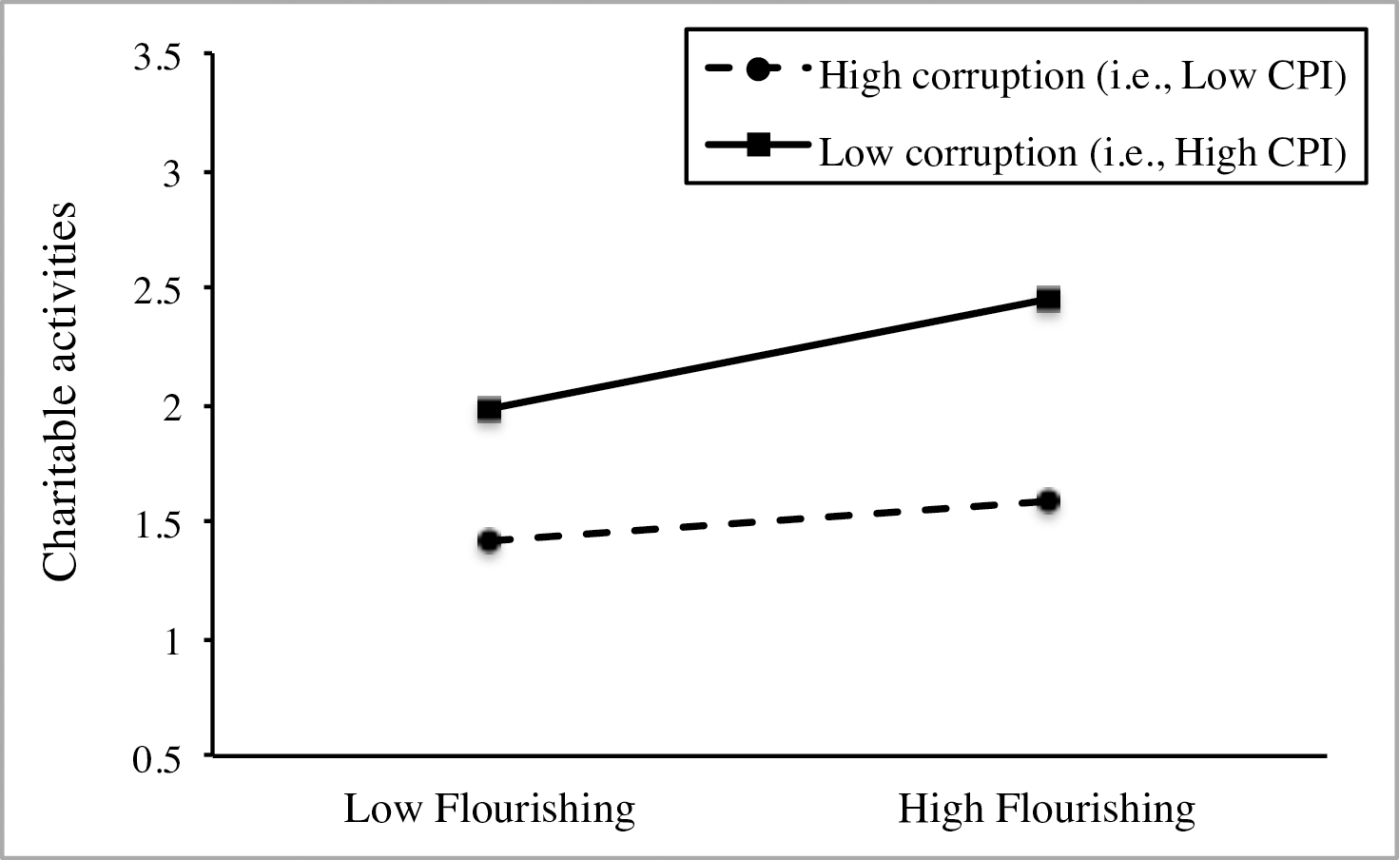
Cross-level interaction between flourishing and CPI on charitable activities in Study 2.

Model 4 indicated that the relation between flourishing and charitable activities differs between countries and justified the investigation into a cross-level moderation effect. Model 5 showed that the main effect of national-level CPI on charitable activities was not significant when controlling for individual-level differences on flourishing and age, and national-level differences on GDP per capita and GINI Index. Finally, Model 6 supported the moderating effect of national-level CPI on the relation between flourishing and charitable activities. The significance of the positive interaction term implies that (a) the effect of flourishing on charitable activities depends on CPI and (b) the coefficient for flourishing is larger in countries with higher CPI (i.e., less corruption), as depicted in Fig 3. Particularly, the calculated explanatory power indicated that the moderating effect of national-level CPI accounted for 58.1% of the slope variance in the relation between flourishing and charitable activities. Further simple slope analysis showed a stronger positive association between flourishing and charitable activities in countries high in CPI (i.e. low corruption, simple slope *B* = .409, *p* < .001) in comparison to countries low in CPI (i.e. high corruption, simple slope *B* = .157, *p* = .003).

These findings demonstrate a close replication of the results in Study 1, which ultimately lends more support to our Hypothesis 1. In particular, the cross-level interaction effect of flourishing and corruption was significantly associated with charitable activities. More specifically, and consistent with our findings in Study 1, flourishing showed a stronger positive effect on engagement in charitable activities for individuals who were living in less corrupt countries, as predicted in Hypothesis 1.

#### Flourishing, CPI and helping close others

Table 6 presents the results of the MLM examining the relation between flourishing, CPI and helping close others. The likelihood ratio test showed that the − 2LL difference between Model 1 and a model without the random component of the intercept equaled 155,210.009–152,204.116 = 3,005.893. The two models differed by only a single degree of freedom, for *χ*^2^ (1) = 3,005.893, *p* < .001, which suggests that participants from different countries vary significantly on the extent to which they engage in helping their close others, on average. The ICC was .031, indicating that only 3.1% of the total variance of helping close others was due to between-country variation. While some would argue against the necessity of MLM in cases where ICC is sufficiently close to zero, the results of the likelihood ratio test corroborated that participants’ engagement in helping their close others significantly differs between countries. Moreover, even small values of ICC have proven sufficiently able to invalidate hypotheses tests and confidence intervals when MLM is not used, specifically in large groups [89]. In addition, as our hypothesis concerns the interaction between a level-1 unit variable (i.e., flourishing) and a level-2 unit variable (i.e., CPI), the MLM provides the most appropriate approach for testing our hypothesis [90].

**Table 6:** Parameter estimates for the six models examining the relation of helping close others on flourishing and CPI in Study 2.

		Model 1	Model 2	Model 3	Model 4	Model 5	Model 6
Fixed components							
Intercept	*γ*_00_	5.138***	5.133***	5.141***	5.140***	5.185***	5.171***
Flourishing (F)	*γ*_10_		.743***	.756***	.691***	.694***	.715***
Age	*γ*_20_			.004***	.004***	.004***	.004***
CPI	*γ*_01_					.003	.007*
GDP per capita	*γ*_02_					.000	.000
GINI Index	*γ*_03_					.005	.006
F × CPI	*γ*_11_						-.005**
Variance of random components							
	*τ*_00_	.038**	.039**	.039**	.039**	.028*	.023**
	*τ*_11_				-.025**	-.019*	-.012*
	*τ*_01_				.022**	.022**	.012*
	*σ*^2^	1.173	.999	.991	.976	.976	.976
Model fit							
Model deviance (–2LL)		152204.116	144037.582	143111.627	142378.339	142364.789	142357.555
Model Δ*χ*^2^			8166.534***	925.955***	745.288***	13.550**	7.234**
*Δdf*			1	1	2	3	1
Pseudo *R*^*2*^		0	.143	.149	.162	.171	.175
Explanatory Power							.368

Notes. CPI = Corruption Perception Index (higher values = lower corruption).

* *p* < .05.

** *p* < .01.

*** *p* < .001

**Fig 4:**
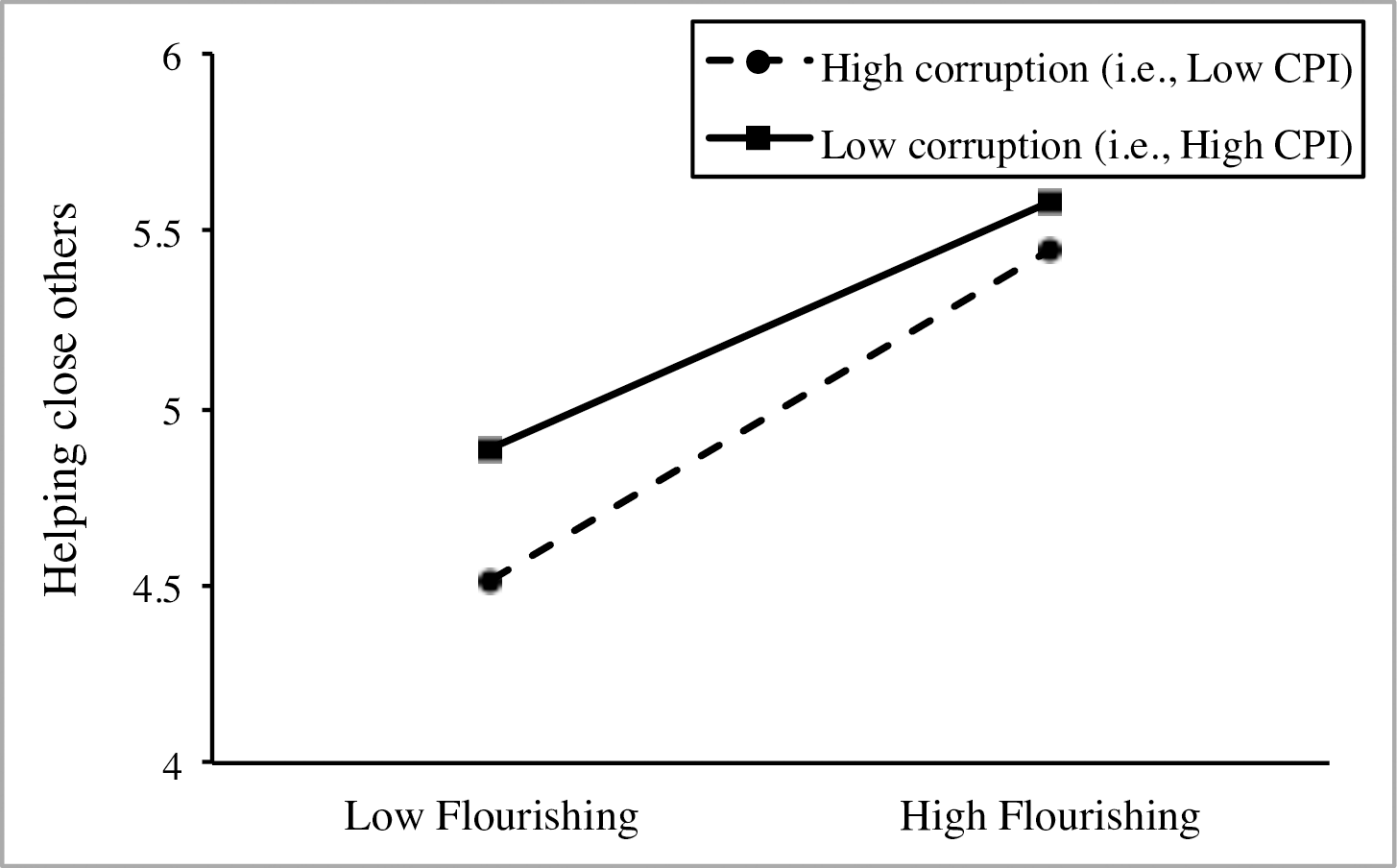
Cross-level interaction between flourishing and CPI on helping close others in Study 2.

Model 2 indicated that flourishing had a strong positive association with helping close others, which remained significant after controlling for the main effect of participants’ age (i.e., Model 3). Model 4 indicated that the relation between flourishing and helping close others differs between countries and justified the investigation of a cross-level moderation effect. Model 5 showed that national-level CPI had a significant main effect on helping close others when controlling for individual-level differences on flourishing and age, and national-level differences on GDP per capita and GINI Index. Finally, Model 6 supported the moderating effect of national-level CPI on the relation between flourishing and helping close others. The significance of the negative interaction term implies that (a) the effect of flourishing on helping close others depends on CPI and (b) the coefficient for flourishing is smaller in countries with higher CPI (i.e., less corruption), as depicted in Fig 4. Specifically, the calculated explanatory power indicated that the moderating effect of national-level CPI accounted for 36.8% of the slope variance in the relation between flourishing and helping close others. Further simple slope analysis showed a stronger positive association between flourishing and helping close others in countries low in CPI (i.e. high corruption, simple slope *B* = .821, *p* < .001) in comparison to countries high in CPI (i.e. low corruption, simple slope *B* = .611, *p* < .001).

With regard to Hypothesis 2, our findings in Study 2 supported that the cross-level interaction effect of flourishing and corruption was significantly associated with helping close others. As we predicted, flourishers who were living in more corrupt countries reported greater engagement in helping their close others compared to flourishers living in less corrupt countries.

## General discussion

Across two studies, we generally found that corruption at the national level influences flourishing individuals’ engagement in prosocial behavior. Specifically, Studies 1 and 2 consistently showed that the positive effect of flourishing on engagement in charitable activities was stronger for individuals who were living in less corrupt countries, as predicted in Hypothesis 1. Study 1 further revealed a similar finding with regard to flourishers’ likelihood of helping distant others. That is, flourishers living in less corrupt countries are more likely to help distant others, as predicted in Hypothesis 1. Moreover, Study 2 indicated that corruption also moderates the relation between flourishing and helping close others. As predicted in our Hypothesis 2, though, flourishers who were living in more corrupt countries reported greater engagement in helping their close others compared to flourishers living in less corrupt countries. Below, we discuss the theoretical contributions of this research and the limitations of our work that may inform future studies. Lastly, we note the practical implications that our findings may have for policymakers.

### Theoretical contributions

The present research contributes to the existing literature in multiple ways. First, our research is one of the few attempts at investigating the broader consequences of flourishing beyond the individual-oriented benefits. Our study thus extends and critically qualifies previous findings, which have shown a positive link between flourishing and engagement in prosocial behaviors, such as making charitable donations, helping others, and exercising organizational citizenship behavior (e.g., [13,91,92]).

Second, we explored the amorality of the flourishing concept, and in doing so, highlight that contextual sources of moral guidance are important to facilitating the prosocial outcomes of a flourishing life. This unique perspective offers a novel perspective for investigating the relation between flourishing and the broader world. Whether flourishers’ journey toward self-realization happens egoistically or in accord with others’ well-being, we argue, is somewhat affected by situational factors. Given our findings, the positive social outcomes of flourishing (e.g., prosocial behaviors) are more likely to occur among flourishers living in more ethical (e.g., less corrupt) societies, as those individuals may perceive social norms to encourage the consideration of others’ interests and well-being.

Third, while the existing research has mostly focused on the direct effects of corruption [31,32,34], the present research uniquely exposed the moderating effect of corruption on the relationship between personal characteristics and prosocial behaviors. Our findings thus accord with an emerging line of research suggesting that a person-situation interaction approach would more accurately capture the variations in socio-moral behaviors (e.g., [28,93]). While this approach is commonly utilized in the business ethics domain (see [57] for a meta-review), its implications for predicting prosocial behaviors at the societal level has remained mostly unnoticed. Particularly, we suggest that the use of national indices of corruption (e.g., Transparency International’s CPI), established by independent experts and business institutions, answers recent calls for alternative and direct measures of situational ethics that transcend individual perceptions [94].

Fourth, the present research took a preliminary step in asserting that ‘who you help matters’. The literature does suggest that helping close others (or in-group members) is more related to the helper’s well-being than helping distant others (or out-group members) [15,16]. Similarly, our findings indicated that there is a stronger positive association between flourishing and prosocial behaviors toward close others (Studies 1 and 2; also see Tables 1 and 4). Further research suggests that prosocial behaviors toward different people (in-group vs. out-group members) happen for distinct, and even opposing, reasons. That is, researchers suggest that collective motives, which may be perceived as egoistic [95], typically drive in-group helping [96]. On the other hand, the combination of inner convictions and general moral principles is more likely to contribute to out-group helping [97].

While the present research did not attend to the underlying motivations for prosocial behavior, our findings lend some support to the above view. On the one hand, we argue that corruption normalizes egoistic attitudes and behaviors, which can motivate helping close others. On the other hand, corruption leads to the belief that acting according to moral principles is not widely praised and practiced in a given society, and thus deters the flourisher’s prosocial behavior toward distant others. Altogether, the present results encourage a continuing focus on differentiating between flourishing’s social outcomes (i.e., for in-group versus out-group members) and uncovering their underlying mechanisms.

### Strengths and limitations

In general, the present research revealed a significant cross-level interaction between flourishing and corruption at the national level that predicted different examples of prosocial behavior. However, it is worthwhile to consider the statistical power and effect sizes of our findings. Based on Hox’s [98] suggestions, obtaining an acceptable level of power in MLM requires a sample of at least 30 groups with at least 30 members in each. Our samples of 50,504 participants from 23 countries (used in Study 1) and 56,835 participants from 29 countries (used in Study 2) speak to a sufficient level of power.

To tackle the issue of effect size, we followed suggestions to report both pseudo *R*^2^ and explanatory power for cross-level interaction terms [84]. In Study 1, we found considerable increases in pseudo *R*^2^ from the null model to Model 6 (i.e., the full model including the interaction term between flourishing and corruption) in predicting both charitable activities (i.e., from 0 to 10.3 percent; see Table 2) and helping distant others (i.e., from 0 to 10.8 percent; see Table 3). In Study 2, pseudo *R*^2^ increased from 0 in the null model to 6.1 percent in Model 6 in predicting charitable activities and 17.5 percent in helping close others (see Tables 5 and 6). Furthermore, cross-level interaction terms between flourishing and corruption yielded substantial explanatory powers of 35.8%, 25.0%, 58.1%, and 35.8% across our analyses, respectively (see Tables 2, 3, 5 and 6). While we acknowledge that there is no current agreement over the best practice for calculating and interpreting effect sizes in MLM (see [84,85,99] for discussions), we think that these results collectively imply that the interaction terms possessed reasonable strength.

Nonetheless, our methodological approaches featured some limitations. First, because our studies relied on correlation analyses, these findings may raise the question of alternative explanations. This is particularly relevant given the literature’s current ambiguity over the causal order of the relationship between well-being and prosocial behavior. Specifically, prior research has both theoretically and empirically advanced that the relation between well-being and prosociality can happen in both directions (cf., [75,100–102]). These considerations further levy that there might be a positive loop between flourishing and prosocial behaviors [103,104]. Despite the fact that interaction effects are very unlikely to be explained by reverse causality, we readily encourage future research to use alternative designs (e.g., experimental or longitudinal) that could potentially unearth the causal inferences.

Second, the use of self-reported data (with the exception of national indices of corruption, GDP per capita, income inequality and individualism) may also raise concerns over the common method/source bias. However, we believe that using self-ratings was appropriate because assessing our variables of interest required full knowledge of participants’ private behaviors, perceptions, intentions, attitudes, and life choices—all of which can be more accurately measured with self-reports [105]. Also, the concern over common method bias is somewhat mitigated by our focus on interaction terms [106]. Indeed, some researchers have suggested that any single-source bias may only lead to an underestimate of interactions [107,108]. Nevertheless, we willingly recognize the preference for multi-source, multi-stage studies that can produce increased confidence in the current conclusions.

Besides, our study is also theoretically limited in regards to the operationalized definition of flourishing. Flourishing, mostly rooted in profound philosophical thoughts, does not easily lend itself to clear-cut scientific definitions such as preferred by contemporary psychology [42,109]. Hence, like many other concepts, there is no common currency for definition of flourishing [43]. This definitional problem is, also, potentiated by the common ‘elemental realism’ stance in defining flourishing—an assumption that “one can know the true nature of reality and objectively discover the elements of which it is composed” [110]. As such, providing a single definition of flourishing with a certain set of elements that appeals to all researchers seems a far-fetched mission. Therefore, while we based our empirical quest in the present research on one of the established operationalizations of flourishing [2], we admit that these findings may not necessarily be extended to the other definitions of flourishing in the field (e.g., [5,43,111]). Nevertheless, if science wants to have an integrated informed discussion, we believe that the future psychological research seems well advised, perhaps also through a constructive incorporation of the insights from other disciplines, to provide a more comprehensive definition and operationalization for the notion of flourishing.

### Concluding remarks

In recent decades, positive psychologists have pioneered many invaluable efforts aimed at increasing policymakers’ awareness of the importance of well-being [112,113]. Such efforts hinge on a central message: Indicators of personal well-being, on top of common indicators of economic growth (e.g., GDP), “provide crucial information for policy-makers interested in fostering the welfare of citizens” [53]. To this end, scholars argue that measuring and developing flourishing populations is not only a way to bolster individuals’ quality of life, but also to augment societal well-being and prosperity [1,36,114]. The present research, however, added a layer of nuance to this argument. Particularly, our research made the case that the personal benefits of flourishing, if not ushered by societal moral values, would not automatically prompt prosocial behaviors toward distant others.

Thus, while we support attempts at increasing personal flourishing at the national level, we also acknowledge that a society of flourishers may not lead to the highest levels of “citizenship responsibility, nurturance, altruism, civility, moderation, tolerance, and work ethics”—all the moral characteristics that constitute the central premises of positive psychology [37]. Policymakers should therefore treat societal indicators of moral values with at least the same importance as personal flourishing.
